# Implementation science should give higher priority to health equity

**DOI:** 10.1186/s13012-021-01097-0

**Published:** 2021-03-19

**Authors:** Ross C. Brownson, Shiriki K. Kumanyika, Matthew W. Kreuter, Debra Haire-Joshu

**Affiliations:** 1 Prevention Research Center, Brown School at Washington University in St. Louis, 1 Brookings Drive, Campus Box 1196, St. Louis, MO 63130 USA; 2 Department of Surgery, Division of Public Health Sciences, and Alvin J. Siteman Cancer Center, Washington University School of Medicine, Washington University in St. Louis, St. Louis, MO 63130 USA; 3 Department of Community Health and Prevention, Drexel University Dornsife School of Public Health, 3215 Market Street, Philadelphia, PA 19104 USA; 4 Health Communication Research Laboratory, Brown School at Washington University in St. Louis, 1 Brookings Drive, Campus Box 1196, St. Louis, MO 63130 USA; 5 Center for Diabetes Translation Research and Center for Obesity Prevention and Policy Research, Brown School at Washington University in St. Louis, 1 Brookings Drive, Campus Box 1196, St. Louis, MO 63130 USA

**Keywords:** Implementation science, Equity, Health inequities

## Abstract

**Background:**

There is growing urgency to tackle issues of equity and justice in the USA and worldwide. Health equity, a framing that moves away from a deficit mindset of what society is doing poorly (disparities) to one that is positive about what society can achieve, is becoming more prominent in health research that uses implementation science approaches. Equity begins with justice—health differences often reflect societal injustices. Applying the perspectives and tools of implementation science has potential for immediate impact to improve health equity.

**Main text:**

We propose a vision and set of action steps for making health equity a more prominent and central aim of implementation science, thus committing to conduct implementation science through equity-focused principles to achieve this vision in U.S. research and practice. We identify and discuss challenges in current health disparities approaches that do not fully consider social determinants. Implementation research challenges are outlined in three areas: limitations of the evidence base, underdeveloped measures and methods, and inadequate attention to context. To address these challenges, we offer recommendations that seek to (1) link social determinants with health outcomes, (2) build equity into all policies, (3) use equity-relevant metrics, (4) study what is already happening, (5) integrate equity into implementation models, (6) design and tailor implementation strategies, (7) connect to systems and sectors outside of health, (8) engage organizations in internal and external equity efforts, (9) build capacity for equity in implementation science, and (10) focus on equity in dissemination efforts.

**Conclusions:**

Every project in implementation science should include an equity focus. For some studies, equity is the main goal of the project and a central feature of all aspects of the project. In other studies, equity is part of a project but not the singular focus. In these studies, we should, at a minimum, ensure that we “leave no one behind” and that existing disparities are not widened. With a stronger commitment to health equity from funders, researchers, practitioners, advocates, evaluators, and policy makers, we can harvest the rewards of the resources being invested in health-related research to eliminate disparities, resulting in health equity.

Contributions to the literature

- There is an urgent call to address issues of equity, health, and social justice in the USA—driven in part by greater awareness of striking increases in economic inequality and the visibility and impacts of structural racism and associated societal problems.
- Implementation science is an area of research with high potential to accelerate progress toward achieving health equity goals in both public health and healthcare.
- We provide 10 recommendations to advance health equity as a priority of implementation science to address challenges in building the evidence base, developing new measures and methods, and addressing context.
- Actions to address ours and other recent recommendations are likely to further health equity and implementation science.

## Background

There is growing urgency to tackle issues of equity and justice [1, 2], which is driven by greater awareness of decades-long increases in income and wealth inequality [3] and the visibility and impacts of structural racism and associated societal problems [4]. Longstanding socioeconomic and racial/ethnic disparities in numerous health outcomes are prominent among these societal challenges and are now exacerbated by the impacts of the COVID-19 pandemic [5]. The focus on equity is spreading quickly across many sectors and in very public ways [6]. A renewed focus on social and structural determinants of health, including racism and discrimination, is also advancing within the scientific community [7].

Health equity is a framing that moves away from a deficit mindset of what society is doing poorly (disparities) to one that is positive about what society can achieve [8]. Beginning in Europe, there has been growing attention on health equity—the commitment to reduce and ultimately eliminate health disparities [9–12]. In the UK, Whitehead framed health inequities as not only avoidable but also unjust [13]. Braveman and colleagues pointed out that achieving health equity involves closing health gaps between those less and those more advantaged while also improving the health of the population overall [9]. Health inequity is addressed through a range of approaches: changing large-scale policies to offset historical injustices, changing policies and practices within healthcare settings, and changing organizational or community contexts that influence health.

A clear distinction is needed between systems, programs, or policies that are *equitable*, that is, accounting for systematic social disadvantage and injustice, and those that might seem fair because they use the language of *equality*. Viewing everyone equally assumes, incorrectly, that all population groups have similar circumstances, resources, and opportunities for achieving good health. For example, changing policies that target longstanding injustices fosters equity by improving social and economic conditions, such as poverty and the opportunity structure for education, housing, employment, and access to healthcare.

Implementation science is an area of research with particular potential to accelerate progress toward achieving health equity goals [14]. Implementation science seeks to understand and influence how scientific evidence is put into practice for health improvement [15]. It offers an explicit response to the decades of scientific progress that generally have not translated into equitable improvements in population health [16]. Applying the perspectives and tools of implementation science has potential for immediate improvement of health equity. Moreover, a greater emphasis on health equity could attract new and more diverse scientific talent to fully invest in implementation science solutions.

Although a focus on disparities and/or health equity has long been an emphasis of implementation science, a more explicit priority on health equity is timely [14, 17–20]. Elements of health equity are now being more prominently considered in implementation science models (aka, frameworks, theories), in the context of implementation of interventions, and in study methods. Recent conceptual models, such as the Health Equity Implementation Framework [20], have begun to examine more deeply a broad array of social determinants of health, rather than simply adding a disparities component to the large set of existing implementation science frameworks [21]. Incorporating a strong equity focus in implementation science requires not only a deliberate emphasis on the needs, culture, and history of the populations and communities [22, 23], but also more critical analyses and deeper understanding of systems and policies, including care delivery and provider attitudes from which inequities might arise. Equity-centered research relies on meaningful engagement and partnership with multiple stakeholders, builds on existing resources, develops shared goals, and integrates knowledge and action that lead to a fairer distribution of power and the benefits of an intervention for all partners [23–25].

In this article, we propose a vision for making health equity the highest priority in implementation science, and thus a central indicator of the field’s success. This means actively seeking and positively addressing issues of diversity and disadvantage to improve the relevance, effectiveness, equity, and impact of implementation science. We identify and discuss challenges in current approaches to addressing health disparities in the context of implementation science and provide recommendations to move the field forward to achieve health equity. While our article is focused largely on public health and health equity challenges in the USA, we draw upon literature and experiences from other regions of the globe.

## Key challenges

Here, we address three important challenges for health equity in implementation science. We propose that each can be overcome, and provide a set of recommendations with specific steps to address them.

To identify relevant literature for this article, a review of reviews was conducted using searches for English-language documents published between January 2015 and February 2021. Electronic databases searched included PubMed, Google, and Google Scholar. Keywords included “health equity,” “heath disparities,” “health inequalities,” “implementation,” and “social determinants of health.” Following screening of titles and abstracts, full papers were reviewed and examined for the following information: focus of the study; type of review (i.e., narrative review, review of systematic reviews, scoping review, systematic review, umbrella review); and selected findings of relevance to health equity and implementation science.

### Limitations of the evidence base

There are at least two important limitations of current scientific evidence that must be overcome to achieve health equity goals: (1) too few evidence-based interventions (EBIs) adequately include a systems approach or address upstream social determinants and (2) the lack of diversity in study samples and settings limits applicability of research findings in ways that unintentionally benefit some populations more than others, potentially exacerbating health inequities.

In implementation research, an EBI is central [26]—often addressing some well-established risk factor (e.g., tobacco use, lack of cancer screening) [27, 28]. However, the origins of many risk behaviors and exposures are shaped by adverse social determinants of health and root causes of inequities (e.g., structural racism, unjust allocation of power and resources). Even though we have a deep literature on the importance of social determinants [8, 10, 29, 30], most repositories of EBIs are organized around downstream diseases and risk factors, with inadequate attention to upstream factors and solutions [31].

Approaches for developing EBIs characteristically follow a reductionist tradition, where the objective is to reconstruct reality by its linear, cause and effect parts [32], not acknowledging or attending to broader systems in which the risk behavior is embedded [33]. Typically, EBI deployment involves developing and testing an intervention by researchers in a specific population, identifying discordance between where and with whom the EBI was originally tested and a new setting and population of interest (contextual differences), and then adapting the EBI to fit [34]. A more practice-based, systems approach is needed for achieving equity. For example, a health equity approach recognizes that race-based discrimination through one system (e.g., housing) is reinforced in other interlocking systems (e.g., transportation, education) and identifies how these systems can undercut the effectiveness, in real-world practice, of an intervention developed in a best-case, controlled efficacy study. A systems-based approach identifies the leverage points within and across societal sectors with the highest potential for impact on health equity [35, 36].

There is also evidence for an “inverse prevention law,” which suggests that those in most need of benefiting from an EBI may be the least likely to receive it [37]. When an EBI improves health across the general population, it may have an unintended consequence of increasing health inequities for some groups (e.g., low-income populations, certain minority groups) who were less likely to be reached or reached effectively [37–39]. Widening of disparities is a clear indication of the failure of science, practice, and policy to adequately achieve equity. In a review of public health interventions in high-income countries, Lorenc and colleagues found that certain interventions (media campaigns, workplace smoking bans) showed evidence of increasing inequities affecting lower socioeconomic groups [37]. In a policy-focused umbrella review, Thomas and colleagues studied a wide range of policy approaches across seven public health areas: tobacco, alcohol, food and nutrition, reproductive health services, the control of infectious diseases, the environment, and workplace regulations [39]. While most policies were shown to either reduce inequities or were neutral toward inequities, some appear to increase inequities (e.g., folic acid mass media campaigns, low emission zones in cities). Implementation of broad policy approaches thought to be universal may require adjustments to aspects of disadvantaged settings and populations to achieve equitable effects.

### Underdeveloped measures and methods

The measures and methods for implementation science are evolving but to date with a limited emphasis on equity and, therefore, a lack of methods that are sensitive to equity issues. For example, upstream interventions which are often focused on policy changes are likely to decrease inequities if appropriately designed [37], but a review of measures of policy implementation found that none of the 170 measures used in a diverse set of studies had an explicit focus on equity [40].

Policy and other interventions that address fundamental health equity issues should be studied with the strongest designs possible. In some cases, the biomedical “gold standard” designs (e.g., the randomized controlled trial) can be used, including cluster-randomized trials [41] and stepped-wedge designs [42]. There is growing literature on how to conduct randomized trials that are equity-relevant [43–45]. In other cases, particularly when the independent variable (e.g., a policy) cannot be randomized, non-randomized designs and methods are appropriate (e.g., time-series designs, quasi-experiments, natural experiments, difference in difference studies) [41, 46]. This range of study methods can help with understanding and addressing policies and equity-related issues in various contexts [41]. Also relevant are pragmatic trials that address issues of importance to key stakeholders by conducting research in real-world conditions, seeking to enhance external validity and other information relevant to transferability to other settings [47]. Such pragmatic approaches facilitate equity-driven implementation science due to their ability to assess multilevel impacts and an emphasis on who benefits and who does not benefit from an EBI [48].

### Inadequate attention to context

Context is a central feature of implementation science, yet it is frequently poorly defined or goes unreported [49]. Often context relates to characteristics of a priority population of focus or the setting within which an EBI is being delivered [50, 51]. Failure to fully account for context limits the applicability and generalizability of study findings to different populations, settings, and time periods [51]. Gaps in our current approaches to context include (1) inadequate attention to macro forces that shape implementation and (2) a need to re-visit the role of EBI adaptation.

To advance equity, we need to more fully account for (macro-level) historical, cultural, economic, and political forces that shape implementation in low-resource settings and communities within the USA and in other countries [14, 52]. When implementing or scaling up an EBI, contexts should be conceptualized in terms of aspects of the intervention and its delivery that are likely to differ from those of the original study population in populations affected by inequities, focusing on those factors that are likely to influence intervention uptake, salience, and effectiveness. First, during implementation planning, a community assessment should account for historical, cultural, and system factors such as structural racism and mistrust of health systems [53]. Second, selection of an EBI is a critical part of implementation and should avoid the assumption that any EBI is good for anyone in any context [54]. And third, it is useful to consider contextual issues across all levels of a socio-ecological framework (individual, interpersonal, organizational, community, policy) [52]. Table 1 summarizes reviews of equity-relevant studies describing essential implementation contextual elements for interventions among disadvantaged populations and low-resource settings [55–59].

**Table 1:** Summary of recent reviews of implementation of interventions addressing health equity and social determinants of health

First author Focus of study	Year of publication	Type of review	Selected findings
Pinto [55] PrEP implementation in the USA	2018	Narrative review	Implementation is not often addressing key barriers: • Within healthcare systems, the lack of communication about, funding for, and access to PrEP • The lack of attention to the intersection between PrEP-stigma, HIV-stigma, transphobia, homophobia, and disparities across gender, racial, and ethnic groups
Yapa [56] Implementation in resource-poor countries and communities	2018	Narrative review	Among existing implementation science in resource-poor countries and communities, three key opportunities were identified: • Intervention and methods innovations may thrive under constraints due to higher creativity when choices are restricted • Reverse innovation transferring novel approaches from resource-poor to research-rich settings will gain in importance • Policy makers in resource-poor countries tend to be open for close collaboration with scientists to inform national and local policy
Alonge [57] Implementation in low- and middle-income countries	2019	Systematic review	• Most of the studies were not conducted under routine conditions for management and financing • Most studies do not describe implementation characteristics completely; more complete descriptions are needed on implementation strategies, implementation variables, and the context under which implementation occurs • More rigorous and adaptive research designs are needed to address how to scale-up and sustain interventions
Harding [58] Implementation in Indigenous communities	2019	Systematic review	Among studies of indigenous communities in Australia, Canada, New Zealand, or the USA, four implementation themes were common: • Studies showed high levels of community engagement • From the culture-centered approach, most studies reflected moderate to high levels of community voice/agency • Most studies addressed systems thinking • Approximately 40% of studies included high levels of end user (e.g., policy makers, tribal leaders) engagement reflective of integrated knowledge translation
Wali [59] Community engagement in Indigenous populations	2021	Scoping review	• Key themes included adapting for the local cultural context and the inclusion of community outreach • Despite the claimed use of participatory research methods, only 6 studies involved community members to identify the area of priority and only 5 used Indigenous interviewing to provide feedback

Contextual conditions drive adaptation—e.g., how an EBI needs to be adapted for a population different from the one with which it was originally developed [60]. However, it is worth re-examining the very concept of adaptation, which one could argue illustrates the relative failure of science to develop relevant solutions for disadvantaged populations. Too often, adaptation is an exercise in retrofitting EBIs to underserved populations and under-resourced settings. In a true equity approach to implementation, a goal might be a steady reduction in the need for EBI adaptation when more and more EBIs are developed in circumstances with the least, rather than the most resources.

## Recommendations

To tackle these and related challenges, we offer 10 recommendations (Table 2). Each is directly linked to the challenges noted and is based on the existing literature and the authors’ experiences.

**Table 2:** Recommendations to advance health equity within implementation science

Domain	Recommendation	Core elements	Actors^**a**^
*Evidence base*			
	1. Link social determinants with health outcomes	• Build literature linking social determinants with health outcomes of importance to key stakeholders (e.g., funders) • Build the literature on implementation processes in low-resource settings • Identify opportunities to address social risk in primary care • Describe the role of social determinants as moderators of behavior change • Apply equity-relevant guidelines and evidence frameworks	• Funders • Researchers
	2. Build equity into all policies	• Incorporate health and equity consideration in policy decisions across sectors (Equity in All Policies) • Analyze barriers to change with an equity focus • Frame and communicate policy information in new ways (e.g., framing for audience segments, use of narratives)	• Advocates • State and local practitioners • Policy makers
*Methods and measures*			
	3. Use equity-relevant metrics	• Expand macro-level metrics to focus on upstream indicators to measure progress toward equity in communities • Identify new metrics in studies to address context and historical disadvantage • Apply existing taxonomies (e.g., outcomes developed by Proctor et al.) in an equity context	• Funders • Researchers • State and local practitioners
	4. Study what is already happening	• Describe how end users experience implementation • Work with practitioners and policy makers to conduct natural experiments • Enhance the role of equity in tailored implementation	• Funders • Researchers • Program evaluators
	5. Integrate equity into models	• Identify the focus of existing models regarding equity and related gaps, social determinants, and stakeholder engagement • Identify methods for fully integrating equity into existing models • Use interactive webtools to increase the focus on equity	• Researchers • Program evaluators
	6. Design and tailor implementation strategies	• Apply lessons from previous studies of implementation and scale-up • Enhance the explicit focus on equity among implementation strategies • Test novel strategies at multiple levels • Enhance the role of adaptive designs in development of equity-relevant implementation strategies	• Researchers • Program evaluators
*Context*			
	7. Connect systems and sectors outside of health	• Establish the premise that justice across societal sectors is essential • Conduct more disease-agnostic interventions • Apply models and methods from systems science	• Advocates • Funders • Researchers • State and local practitioners • Health system leaders
	8. Engage organizations, internally and externally	• Internally, assess climate and culture with an equity focus • Evaluate existing programs and policies regarding their equity impacts • Externally, bring on new equity partners, share power and decision-making, and break down funding silos	• Researchers • State and local practitioners • Program evaluators
*Cross-cutting issues*			
	9. Build capacity for equity	• For the “who” of capacity building, increase engagement of persons in trainings from under-represented minority backgrounds • Re-shape the “how” of trainings with an equity lens on the audience, competencies, engagement, and evaluation • Add new settings to expand the “where” of capacity building	• Funders • Researchers
	10. Focus on equity in dissemination efforts	• Provide incentives for researchers to engage with end users in ways to improve dissemination • Engage with equity-focused partners early and often in the research process • Develop new dissemination products that resonate with key stakeholders	• Advocates • Funders • State and local practitioners • Researchers

^a^Individuals, groups, and community partners most likely to take action to address the recommendation

### Improving the evidence base

#### Link social determinants with health outcomes

Many funders of research in public health and healthcare delivery (including those in implementation science) tend to require studies that show effects on traditional clinical and behavioral outcomes (e.g., cancer screening rates, rates of depression, rates of infectious disease) [61]. A clear need for health equity in implementation science is the ability to understand pathways between social determinants of health and outcomes of relevance to various stakeholders, recognizing that the impact may be neither direct nor immediate, but still profound and measurable. There is a substantial and growing body of evidence linking interventions on social determinants of health to a broad range of health outcomes (Table 3) [4, 38, 39, 62–68]. In primary care studies, there are multiple ways in which social determinants of health are increasingly being addressed (e.g., screening for social risk factors, linking patients with local resources) [69]. In addition, it is important to consider social determinants as potential moderators of health behavior change [70].

**Table 3:** Summary of recent reviews of priorities for and impacts of interventions addressing health equity and social determinants of health

First author Focus of study	Year of publication	Type of review	Selected findings
Paradies [62] Racism as a determinant of health	2015	Systematic review (meta-analysis)	• Racism was associated with poorer mental health (*r* = − .23; 95% confidence interval (CI) = − .24, − .21) • Racism was also associated with poorer general health (*r* = − .13; 95% CI = − .18, − .09) and poorer physical health (*r* = − .09; 95% CI = − .12, − .06) • Among physical health outcomes, racism was associated with diabetes (*r* = − .02; 95% CI = − .09, .04)
Purnell [63] Gaps and innovative interventions for health equity	2016	Narrative review	• Identified persistent disparities for cardiovascular disease and cancer risk by race and urban-rural residence • Culturally tailored, *promotora*-based interventions improved mammography screening among Latinas • Use of referrals to community resources to address sociocultural barriers was associated with smoking cessation • A stakeholder-engaged, culturally tailored intervention was effective in controlling blood pressure
Taylor [64] Impacts of investments in social determinants interventions	2016	Narrative review	• Housing support interventions showed reduced healthcare costs and improvements in health outcomes (obesity, diabetes, asthma, HIV) • Nutrition support interventions improved health outcomes (protective for low birthweight and preterm delivery, body weight) • Income support interventions consistently demonstrated a positive impact on health outcomes (infant mortality, disability rates, mental health) • Care coordination and community outreach interventions showed decreased healthcare costs
Asare [65] Social determinants and cancer disparities	2017	Narrative review	• Social and economic factors may negatively affect minority patients’ ability to participate in cancer research • Lack of accessible transportation can restrict access to health and cancer care • Exposure to discrimination may lead to mistrust of elements of this society and suspicion of healthcare systems • The social determinants of health framework posits that all social and economic constructs are interrelated
Bailey [4] Structural racism and health inequities	2017	Narrative review	• Most studies on racism and health have focused on interpersonal racial/ethnic discrimination, with comparatively less emphasis on investigating the health effects of structural racism • Structural racism involves interconnected institutions, whose linkages are historically rooted and culturally reinforced Promising approaches include: • Use of a focused external force that acts on multiple sectors at once (e.g., place-based multisector initiatives such as Promise Neighborhoods) • Disruption of leverage points within a sector that might have ripple effects in the system (e.g., reforming drug policy and reducing excessive incarceration)
Dendup [66] Environmental risk for type 2 diabetes	2018	Systematic review	• Walkability, air pollution, food and physical activity environment, and roadways proximity were the most common environmental characteristics studied • Higher levels of walkability and green space were associated with lower risk of type 2 diabetes • Increased levels of noise and air pollution were associated with greater risk
Suleman [67] Xenophobia and health	2018	Scoping review	• Among individuals living with HIV, xenophobia is a barrier to medical service access • Xenophobia is associated with higher rates of mental health outcomes (e.g., depression, chronic anxiety, psychoses)
Thomson [39] Health policies and inequalities	2018	Umbrella review of systematic reviews	• Results were mixed across the public health domains • Some policy interventions were shown to reduce health inequalities (e.g., food subsidy programs, immunizations) • Some policy interventions had no effect • Some interventions appear to increase inequalities (e.g., 20 mph and low emission zones)
Naik [38] Macroeconomic determinants of health inequalities	2019	Review of systematic reviews	• Policies to promote employment and improve working conditions can improve health and reduce gender-based health inequities • Market regulation of tobacco, alcohol, and food was effective at improving health and reducing inequities (rates of smoking, alcohol use, healthy food consumption) • Privatization of utilities and alcohol sectors, income inequality, and economic crises increase health inequities
Martinez-Cardoso [68] Social determinants of diabetes management	2020	Narrative review	• Diabetes management and care is deterred by housing precarity, food insecurity, poverty, uninsurance and underinsurance, and limited support for immigrants in healthcare systems • Interventions to address diabetes require a more upstream approach to mitigate the drivers of diabetes disparities among immigrants

There are opportunities for researchers to more fully consider social determinants of health as they design studies. To inform future studies and to synthesize existing literature, several useful guidelines and evidence frameworks can be applied. For example, equity and social determinants are included in the APEASE criteria [71], the GRADE Evidence to Decision framework [72, 73], and an expanded version of CONSORT [74–77].

#### Build equity into all policies

Policies, in the form of laws and administrative regulations, have profound effects on population health and health equity. Policy implementation is an under-studied area, particularly in the USA [78, 79]. Research on policy implementation seeks to understand the complexity of the policy process to increase the likelihood that research evidence is a meaningful part of policy decisions. In this section, we focus on “Big P” policies (i.e., laws, administrative rules, and regulations), although in a later section, “small p” policies (i.e., organizational changes, non-governmental professional guidelines) are briefly considered.

Lessons can be drawn from the Health in All Policies (HiAP) movement which recognizes that our greatest health challenges are complex and strongly determined by policy and social determinants [80, 81]. The HiAP approach seeks to incorporate health considerations in decision-making across sectors and policy topics. We propose a new framing as Equity in All Policies (EiAP), in which equity is a primary consideration, not merely one of many considerations. An EiAP approach would include analyzing barriers to policy change, the impacts of policy decisions on equity, both retrospectively and prospectively, with a particular emphasis on aspects of policy design that can privilege or disadvantage certain population groups [39, 82, 83]. Lack of evidence often is not the main barrier to policy action to address equity, more often political will is the biggest challenge [30]. To build political will, new approaches are also needed for framing and communicating the health equity benefits to various segments of policy audiences (e.g., progressive versus conservative) via audience research studies [84].

### Improving measures and methods

#### Use equity-relevant metrics

A public health adage is “what gets measured, gets done” [85]. Most existing measures focus on ultimate outcomes, such as disparities in health status, and do not directly measure factors that lie along the pathway to inequity or equity. Equity measurement should include three elements (1) an indicator of health or a modifiable determinant of health (e.g., living conditions, policies), (2) an indicator of social position (e.g., economic stability, educational attainment), and (3) a method for comparing health or a health determinant across social strata (e.g., a ratio of rates) [86]. Two groups of metrics need to be developed and used: (1) broad equity measures of social determinants of health that could be used to measure progress in communities and (2) measures specific to equity and implementation science for use in research studies.

At a population level, representing the success or failure of implementation, we need to expand our usual surveillance metrics (e.g., behavioral risk factors, mortality) and crude area-level measures (e.g., the Area Deprivation Index) to concentrate more on upstream factors. For example, surveillance systems should track social determinants such as third grade reading literacy, unemployment rates, incarceration rates, and the percentage of households that pay over 30% of their income for housing.

**Fig. 1:**
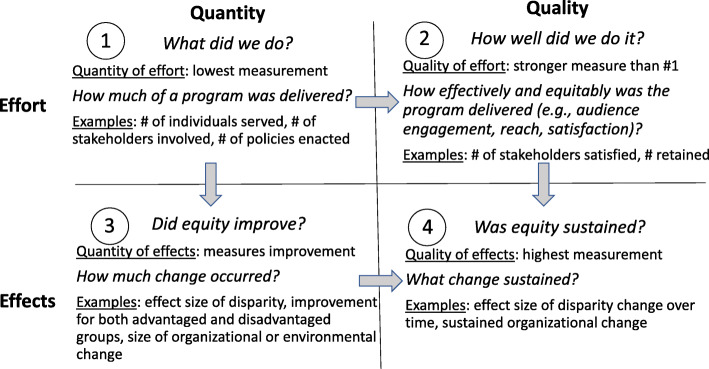
Four categories of measurement for equity and implementation science (adapted from MCH Evidence [91])

Within implementation research studies, we need to go deeper into the underlying causes of disparities, identify new metrics, and include these in our studies. For example, to measure disadvantage, many studies measure household income but few measure household wealth. The Black-White difference in median household income is 1.7-fold yet the difference in Black-White median household wealth is 10-fold [87, 88]. To more fully measure equity, researchers need to develop measures that account for historical or life course disadvantage and metrics within multiple levels that account for the context for implementation [89, 90]. A measurement approach for equity in implementation science assesses both quantity and quality—the simplest measurement occurs in quadrant 1 and the most comprehensive in quadrant 4 (Fig. 1) [91].

As equity-relevant measures are developed and refined, it will be helpful to apply existing taxonomies such as the set of eight outcomes developed by Proctor and colleagues (i.e., acceptability, adoption, appropriateness, feasibility, fidelity, implementation cost, penetration, and sustainability) [92]. Measures and methods within these eight categories will need to be adapted to account for contexts where disparities are developed and maintained (e.g., how to document feasibility in a low-resource setting).

#### Study what is already happening—more practice-based evidence

The importance of context is often devalued relative to the presumed “superiority” of the intervention itself. Our funding mechanisms tend to favor innovation over replication, even when many stakeholders are largely seeking to apply existing knowledge [93]. The research process does not always align with priorities of those experiencing inequities and often moves more slowly than innovations in practice and policy. In a study of implementation of mental health services, Aby found three important themes showing how participants experienced implementation: invisibility (e.g., not enough mental health providers of color), isolation (e.g., separation and lack of collaboration among key stakeholder), and inequity (e.g., feeling tokenized or unwelcome) [94].

To address these issues, it will be useful to place much more emphasis on studying implementation of ongoing health equity projects, often via natural experiments. Experience in low-resource settings shows that novel approaches sometimes thrive under constraints (i.e., creativity can thrive when choices are restricted) [56]. These real-world studies often involve natural experiments [95, 96], which are particularly useful in studying social determinants designed to address inequities and enhance external validity [97]. Multiple state and local agencies across the USA are conducting health equity projects [98]. As an example, the Rhode Island Department of Health supports nine Health Equity Zones across the state to improve socioeconomic and environmental conditions across the state [99]. In Canada, as part of the Canadian Coalitions Linking Action and Science for Prevention (CLASP) initiative, First Nations, Inuit, and Métis community partners brought unique and important community perspectives and relationships to implementation projects [100, 101]. These projects develop so-called “tacit knowledge” or “colloquial evidence” (pragmatic information based on direct experience and action in practice) [102, 103]. They also contribute to the process of tailored implementation, which builds on real-world experiences to identify the most important determinants to address [104, 105].

#### Integrate equity into implementation models

Models provide a roadmap—a systematic structure for the development, management, and evaluation of all parts of a study [21]. While there are over 100 models to guide implementation science research, only a handful explicitly include health equity [20, 90, 94, 106–109]. These frameworks are informed by development and use of a larger set of models on health and equity [110].

To advance equity and implementation science, we need to identify gaps among existing models which in turn can guide model improvement. This analysis could assess (1) whether equity is an explicit focus of models (e.g., Is it an end goal?), (2) the degree to which social determinants are represented, (3) whether a set of core equity constructs could be identified within models, (4) whether models apply equally well to lower and higher resource settings, and (5) the degree of representation of disadvantaged groups and community stakeholders in the model. Model selection and adaptation can benefit by interactive webtools such as *Dissemination and Implementation Models in Health Research and Practice* [111].

#### Design and tailor implementation strategies

Implementation strategies are methods to enhance the adoption, implementation, sustainment, and scale-up of an innovation (often in the form of an EBI) [112]. Multiple taxonomies describe and organize commonly used implementation strategies that can target a range of stakeholders and multilevel contextual factors across different phases of implementation [113, 114]. The design of implementation strategies should be guided by the growing body of evidence, pertinent theories and frameworks, and relevant stakeholders, including those from communities in which health disparities have been identified [113, 115]. Ultimately, the goal is to understand who needs to do what in order to implement and sustain an innovation, what factors are likely to facilitate or impede those changes, and what strategies need to be in place in order to address anticipated or emergent challenges [116, 117]. The implementation and scale-up of the U.K. Diabetes Prevention Programme show the importance of stakeholder engagement, addressing contextual conditions (e.g., staff turnover), and the value of incentives [118].

Development and use of implementation strategies should include explicit consideration of disparities, contextual conditions that may lead to inequitable outcomes (e.g., resources, history), and opportunities to promote equity by carefully designing and/or tailoring strategies. To date, relatively little emphasis has been placed on how well implementation strategies are responsive to health equity needs. There is ample opportunity to examine the extent to which strategies identified in prevailing taxonomies can be leveraged to address determinants of equity, and to develop and test novel strategies at multiple levels (e.g., individual, provider, organization, community) that may promote health equity. Adaptive designs for developing equity-relevant implementation strategies, such as the Sequential Multiple Assignment Randomized Trial (SMART) design [119], are likely to be useful in accounting for changing real-world conditions.

### Giving greater attention to context

#### Connect to systems and sectors outside of health

Many of the most important influences on health status and disparities occur in sectors outside of healthcare and public health (e.g., schools, housing, education, labor) [120]. These settings are crucial for implementation science in at least four ways: (1) they are highly experienced delivering services to underserved populations and thus have deep knowledge of how to do it well, (2) many of these sectors are already delivering exactly the kinds of non-health interventions that address social needs (as described in Table 3), (3) they provide access to high-risk populations where a health intervention might be added to a service (e.g., adding a smoking cessation intervention to services designed to meet social needs), and (4) the setting itself might be the focus for change and a secondary benefit is a health outcome (e.g., lower use of the emergency department among those who receive permanent supportive housing). Often, the missions and cultures of agencies in these sectors do not focus on health [121]. Therefore, our traditional approaches for forming partnerships need to be re-examined and altered [122].

Three principles show promise. First, an underlying premise is that justice is essential to achieving health equity [123, 124]—not only justice in the health sector but justice across all sectors including housing and neighborhoods, safety, education, and economics and employment. Second, we need more “disease-agnostic interventions,” which are structural interventions, often outside the health sector, that address common risk factors that are linked with multiple disparities [125]. And third, systems science approaches that link sectors have been increasingly applied in public health to study and develop EBIs to address areas as diverse as global pandemics, vaccination system, cancer, and obesity [126]. To date, systems approaches have not been widely applied in health equity, although they show promise [127].

#### Engage organizations in internal and external equity efforts

Organizations are one of several central entities of influence in implementation science [128]. They may directly deliver health services or may involve community-level partnerships to influence disparities and population health. While health equity is a high priority for many public health organizations, there is sparse empirical data on the organizational commitment to equity issues and how that commitment is operationalized. For example, in a nationwide survey of U.S. practitioners in state health departments, only 2% reported working primarily on health equity and 9% reported that health equity was one of their multiple priority areas [129].

There are opportunities to more fully address issues in equity and implementation science in organizations [121]—both internally and externally. Within an organization, assessments of climate and culture can be conducted with an equity lens. For example, one could assess the perception of the commitment of leaders to equity; employee attitudes, motivations, performance on equity issues (including the presence of hidden biases [130]); internal policies supporting equity; and the diversity of an organization. Organizations could also evaluate existing programs and policies for their reach and impact on health equity. Externally, organizations can bring on new partners who have a shared commitment to equity, develop organizational policies that share decision-making and power with partners, make equity a stated goal of partnerships, and break down funding silos to address root causes. Equity-driven practice for organizations directs resources for those most in need [131].

### Addressing cross-cutting issues

#### Build capacity for equity in implementation science

Recent reviews of initiatives to build capacity in implementation science have shown a growing number of training opportunities across eight countries [132–134]. Capacity building for implementation science occurs in multiple formats including university degree programs, summer training institutes, workshops, and conferences [133]. Nearly all training programs to date have focused on capacity building among researchers (the “push” for implementation science) with little emphasis on practitioners or implementers (the “pull” for implementation science) [133, 135], with few featuring an explicit focus on equity [136].

A full vision for equity-related training needs to be centered on who is being trained, how they are being trained, and where the work is occurring. To address the “who” element in training, programs need to include a larger percentage of early-career scholars who are from under-represented minority groups [137] and those working in disciplines outside of health. The “how” can include multiple parts including (1) how training is delivered to reach all audiences (including those outside the health sector), (2) whether equity is featured as an explicit part of core competencies, (3) how principles of community engagement are included in training [23], and (4) how progress toward equity is evaluated. The “where” issues include where research is occurring and how diverse communities, which are the settings for studies, are engaged in meaningful ways.

#### Focus on equity in dissemination efforts

Designing for dissemination is defined as “an active process that helps to ensure that public health interventions, often evaluated by researchers, are developed in ways that match well with adopters’ needs, assets, and time frames” [138]. There is a well-documented disconnect between how researchers disseminate their findings and how practitioners and policy makers learn about the latest evidence [93]. Experience in the population or setting of focus also matters—public health researchers with practice or policy experience are over four times more likely to report good or excellent skills in dissemination [139].

Equity-focused dissemination of research findings could include several core elements. At a systems level, funders should provide incentives for researchers to engage in meaningful ways with audiences experiencing disparities (e.g., through requirements for dissemination, supplemental funding). To improve dissemination processes, researchers should engage with equity-focused partners early and often in the research process [140]. Products for dissemination could be improved by refining messages that resonate with key stakeholders and developing communications materials in collaboration with the audience of focus that reflect the images, narratives, and outcomes of interest to populations experiencing disparities.

## Conclusions

Approaches to achieving health equity are critical to ameliorating disparities resulting from social, economic, and racial injustice. Given that implementation science is a relatively young field [141, 142], often focused on narrowly-defined EBIs, the lack of explicit attention on equity is not unexpected. As a new field, it is more malleable and should embrace the challenge of equity, a highly ambitious but critically important responsibility that would unquestionably demonstrate its value and provide an identity distinct from the many disciplines it draws upon.

Our premise is that every project in implementation science should include an equity focus. Equity begins with justice—health differences often reflect social injustices [123]. For some studies, equity is the main goal of the project and a central feature of the research questions, the conceptual model, project activities, and dissemination of findings. In other studies, equity is part of a project but not the singular focus. In these projects, we should, at a minimum, ensure that we “leave no one behind” and that existing disparities are not inadvertently widened.

Our recommendations offer a pathway for advancing health equity through implementation science. The ideas provided are critical but far from a complete “playbook” on what needs to happen and how goals might be accomplished. Along with other recent calls to action [14, 20], we view these as first-generation concepts to immediately address health equity—ideas on which others can further advance and build upon. With a stronger commitment to equity from funders, researchers, practitioners, advocates, and policy makers, we can harvest the rewards of the resources being invested in health-related research to eliminate disparities, resulting in health justice.

## Data Availability

Not applicable.

## Abbreviations

APEASE: Acceptability, Practicability, Effectiveness, Affordability, Side-effects, and Equity
CONSORT: Consolidated Standards of Reporting Trials
COVID-19: Coronavirus disease 2019
EBI: Evidence-based intervention
GRADE: Grading Recommendations Assessment and Development Evidence
HiAP: Health in All Policies
EiAP: Equity in All Policies
SMART: Sequential Multiple Assignment Randomized Trial
U.S.: United States
